# Crystal structure of *cis*-(1,4,8,11-tetra­aza­cyclo­tetra­decane-κ^4^ *N*)bis­(thio­cyanato-κ*N*)chromium(III) bromide from synchrotron X-ray diffraction data

**DOI:** 10.1107/S2056989021001055

**Published:** 2021-02-02

**Authors:** Dohyun Moon, Jong-Ha Choi

**Affiliations:** a Beamline Department, Pohang Accelerator Laboratory, POSTECH, Pohang 37673, Republic of Korea; b Department of Chemistry, Andong National University, Andong 36729, Republic of Korea

**Keywords:** crystal structure, chromium(III), cyclam, thio­cyanate ligand, *cis*-V conformation, bromide anion, hydrogen bonding, synchrotron radiation

## Abstract

The crystal structure of the title complex, *cis*-[Cr(NCS)_2_(cyclam)]Br (cyclam = 1,4,8,11-tetra­aza­cyclo­tetra­decane, C_10_H_24_N_4_), has been determined from synchrotron X-ray data. The asymmetric unit contains one [Cr(NCS)_2_(cyclam)]^+^ cation and one bromide anion. The Cr^III^ ion of the complex cation is coordinated by the four N atoms of the cyclam ligand and by two N-coordinating NCS groups in a *cis* arrangement, displaying a distorted octa­hedral coordination sphere. The Cr—N(cyclam) bond lengths are in the range 2.075 (3) to 2.081 (3) Å while the average Cr—N(NCS) bond length is 1.996 (16) Å. The macrocyclic cyclam moiety adopts the most stable *cis*-V conformation. The crystal structure is stabilized by inter­molecular hydrogen bonds involving the cyclam N—H groups as donor groups, and the bromide anion and the S atom of one of the NCS ligands as acceptor groups.

## Abstract 2

The Cr^III^ ion in the [Cr(NCS)_2_(cyclam)]^+^ cation has a distorted octa­hedral coordination environment with four N atoms of cyclam and two N-bonded NCS groups in a *cis* arrangement. The cyclam ligand adopts the *cis*-V conformation.

## Chemical context   

Compounds containing cyclam (1,4,8,11-tetra­aza­cyclo­tetra­decane, C_10_H_24_N_4_) or its derivatives have a potential inhibitory effect on the replication of the human immunodeficiency virus (HIV) and have the ability to mobilize hematopoietic progenitor stem cells from the bone marrow into the blood (Ronconi & Sadler, 2007 ▸; De Clercq, 2010 ▸; Ross *et al.*, 2012 ▸). In order to develop new anti*-*HIV drugs using transition-metal complexes with the cyclam ligand, at first it is necessary to obtain accurate information about their conformations and crystal packing forces (De Clercq, 2010 ▸). Cyclam has a moderately flexible structure, and can adopt both planar (*trans*) and folded (*cis*) conformations in [Cr*L*_2_(cyclam)]^*n*+^ (*L* = monodentate or bidentate/2) complexes (Poon & Pun, 1980 ▸). There are five conformational *trans* isomers for the macrocycle, which differ in the chirality of the *sec*-NH groups (Choi, 2009 ▸; Jeon *et al.*, 2020 ▸). The *trans*-I, *trans*-II and *trans*-V conformations also can fold to form *cis*-I, *cis*-II and *cis*-V conformers, respectively (Subhan *et al.*, 2011 ▸; Jeon *et al.*, 2020 ▸). Knowledge of the conformation for the macrocyclic ligand including various counter-anions are important factors in developing new highly effective anti*-*HIV drugs (Ronconi & Sadler, 2007 ▸; De Clercq, 2010 ▸; Ross *et al.*, 2012 ▸). Furthermore, the NCS group is inter­esting either as a co-ligand or a counter-anion in transition-metal complexes. As an ambidentate ligand, the NCS group can coordinate either through the N or S atom, and can adopt various bridging modes (Moon & Choi, 2021 ▸).
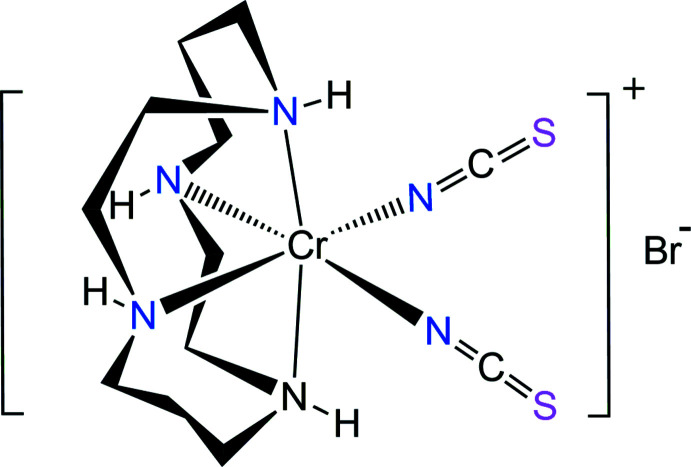


As an extension of our investigations on the coordination chemistry and conformations of Cr^III^ complexes containing the cyclam ligand, one auxiliary bidentate or two monodentate ligands and various anions (Choi *et al.*, 2004*a* ▸,*b* ▸; Choi & Lee, 2009 ▸; Subhan *et al.*, 2011 ▸; Moon *et al.*, 2013 ▸, 2017 ▸; Moon & Choi, 2021 ▸), we describe here the synthesis of a new salt complex, [Cr(NCS)_2_(cyclam)]Br, (I) and its structural characterization by synchrotron single-crystal X-ray diffraction.

## Structural commentary   

**Figure 1:**
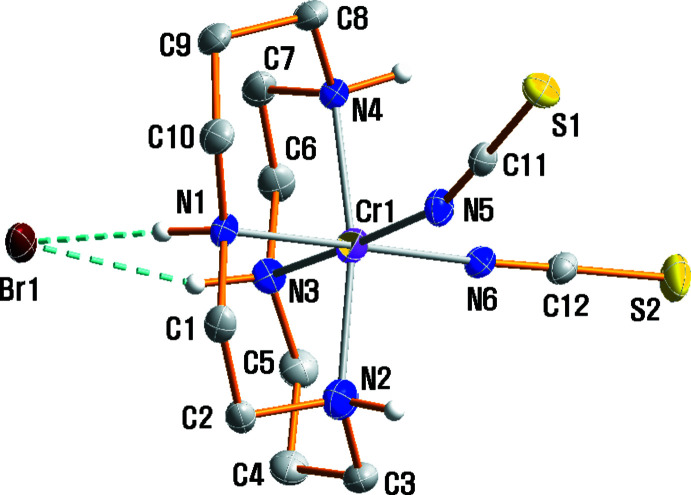
The mol­ecular structure of (I), drawn with displacement ellipsoids at the 30% probability level. Only H atoms of amine groups are shown for clarity.

The mol­ecular structure of (I) with the atomic labelling is shown in Fig. 1 ▸. The crystal structure shows another example of a [Cr(NCS)_2_(cyclam)]^+^ cation but with a different counter-anion than previously reported (Friesen *et al.*, 1997 ▸; Moon *et al.*, 2013 ▸, 2017 ▸; Moon & Choi, 2021 ▸). In general, counter-anionic species play a very important role in coordination chemistry (Martínez-Máñez & Sancenón, 2003 ▸; Fabbrizzi & Poggi, 2013 ▸). The asymmetric unit of (I) comprises one Cr^III^ complex cation, and one Br^−^ anion. In the complex cation, the Cr^III^ ion is coordinated by the nitro­gen atoms of the cyclam ligand that adopts the *cis*-V (*anti*–*anti*) conformation (Subhan *et al.*, 2011 ▸). Two nitro­gen atoms of the NCS groups further coordinate to the central chromium cation in a *cis* arrangement. The Cr—N bond lengths from the donor atoms of the cyclam ligands are in the range 2.075 (3) to 2.081 (3) Å, in good agreement with those determined in *cis*-[Cr(NCS)_2_(cyclam)]SCN [2.0851 (14)–2.0897 (14) Å] (Moon *et al.*, 2013 ▸), *cis-*[Cr(N_3_)_2_(cyclam)]ClO_4_ [2.069 (3)–2.103 (3) Å] (Meyer *et al.*, 1998 ▸), *cis*-[Cr(ONO)_2_(cyclam)]NO_2_ [2.0874 (16)–2.0916 (15) Å] (Choi *et al.*, 2004*a* ▸) and *cis*-[Cr(acac)(cyclam)](ClO_4_)_2_·0.5H_2_O [2.070 (5)–2.089 (5) Å] (acac = acetyl­aceto­n­ate; Subhan *et al.*, 2011 ▸). However, the Cr—N bond lengths of the cyclam ligand in the *cis* conformation are slightly longer than those found in *trans-*[Cr(NCS)_2_(cyclam)]ClO_4_ [2.046 (2)–2.060 (2) Å] (Friesen *et al.*, 1997 ▸), *trans*-[Cr(ONO)_2_(cyclam)]BF_4_ [2.064 (4)–2.073 (4) Å] (De Leo *et al.*, 2000 ▸), *trans-*[Cr(NH_3_)_2_(cyclam)][ZnCl_4_]Cl·H_2_O [2.0501 (15)–2.0615 (15) Å] (Moon & Choi, 2016 ▸) and *trans*-[Cr(nic-O)_2_(cyclam)]ClO_4_ [2.058 (4)–2.064 (4) Å] (nic-O = O-coordin­ating nicotinate; Choi, 2009 ▸). The two Cr—N(NCS) bond lengths in compound (I) average 1.996 (16)Å and are similar to those found in other complexes with this coligand, *viz. cis*-[Cr(NCS)_2_(cyclam)]NCS [1.9846 (13)–2.0071 (13) Å] (Moon *et al.*, 2013 ▸), *cis*-[Cr(NCS)_2_(cyclam)]ClO_4_ [1.981 (4)–1.998 (4) Å] (Friesen *et al.*, 1997 ▸), *cis*-[Cr(NCS)_2_(cyclam)]_2_[Cr_2_O_7_]·H_2_O [1.980 (2)–1.989 (2) Å] (Moon *et al.*, 2017 ▸), *trans-*[Cr(NCS)_2_(cyclam)]_2_[ZnCl_4_] [1.995 (6) Å] (Moon *et al.*, 2015 ▸), and *trans*-[Cr(NCS)_2_(Me_2_tn)_2_]SCN·0.5H_2_O [1.983 (2)–1.990 (2) Å] (Choi & Lee, 2009 ▸). The five-membered and six-membered chelate rings of the cyclam ligand adopt the *gauche* and stable chair conformation, respectively. The fold angle of 95.39 (11)° in the cyclam ligand is similar to those of 98.55 (2), 97.17 (5), 97.03 (2), 95.09 (9), 94.51 (2) and 92.8 (2)° in *cis*-[Cr(ox)(cyclam)]ClO_4_, *cis*-[Cr(NCS)_2_(cyclam)]SCN, *cis*-[Cr(acac)(cyclam)](ClO_4_)_2_·0.5H_2_O, *cis*-[Cr(ONO)_2_(cyclam)]NO_2_, *cis-*[Cr(N_3_)_2_(cyclam)]ClO_4_ and *cis*-[Cr(cyclam)Cl_2_]Cl, respectively (Choi *et al.*, 2004*b* ▸; Moon *et al.*, 2013 ▸; Subhan *et al.*, 2011 ▸; Choi *et al.*, 2004*a* ▸; Meyer *et al.*, 1998 ▸; Forsellini *et al.*, 1986 ▸). The two N-coordinating thio­cyanate ligands are almost linear with N≡C—S angles of 178.8 (3) and 178.9 (3)°. The Cr1—N5—C11 angle of 161.6 (3)° is slightly smaller than that for Cr1—N6—C12 [169.9 (3)°], which may be attributed to the involvement of the S1 atom in a hydrogen-bonding inter­action.

## Supra­molecular features   

**Figure 2:**
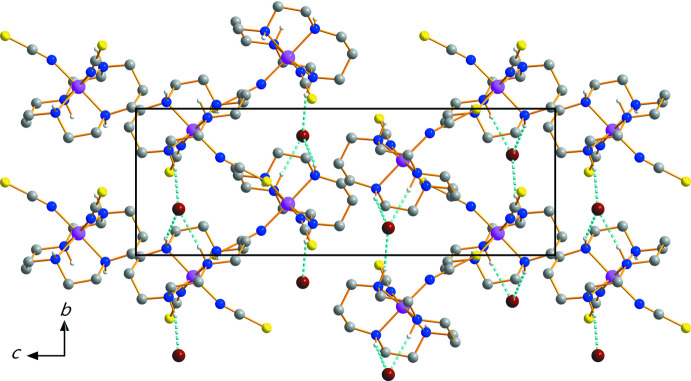
The crystal packing in (I), viewed perpendicular to the *bc* plane. Dashed lines represent N—H⋯Br hydrogen-bonding inter­actions. For clarity, N—H⋯S hydrogen-bonding inter­actions and H atoms bonded to C atoms have been omitted.

The Br^−^ counter-anion remains outside the coordination sphere of the Cr^III^ ion. In the crystal, N—H⋯Br and N—H⋯S hydrogen-bonding inter­actions occur between the N—H groups of cyclam, the Br^−^ anion and the S atom of one of the NCS ligands (Table 1 ▸, Fig. 2 ▸), leading to a three-dimensional network structure. The bromide anion is linked to the [Cr(NCS)_2_(cyclam)]^+^ cation *via* three N—H⋯Br hydrogen bonds. In addition, two [Cr(NCS)_2_(cyclam)]^+^ cations are inter­connected to each other *via* an N4—H4⋯S1^ii^ [symmetry code: (ii) −*x* + 1, *y* + 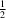, −*z* + 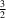] hydrogen bond.

## Database survey   

A search of the Cambridge Structural Database (CSD, version 5.42, November 2020; Groom *et al.*, 2016 ▸) gave 77 hits for a *cis*-[Cr*L*_2_(C_10_H_24_N_4_)]^+^ unit. It is found that *cis*-[Cr(NCS)_2_(C_10_H_24_N_4_)]ClO_4_ (Friesen *et al.*, 1997 ▸), *cis*-[Cr(NCS)_2_(C_10_H_24_N_4_)]NCS (Moon *et al.*, 2013 ▸), *cis*-[Cr(C_2_O_4_)(C_10_H_24_N_4_)]ClO_4_ (Choi *et al.*, 2004*b* ▸), *cis*-[Cr(CH_3_COCHCOCH_3_)(C_10_H_24_N_4_)](ClO_4_)_2_·0.5H_2_O (Subhan *et al.*, 2011 ▸), *cis*-[Cr(NCS)_2_(C_10_H_24_N_4_)]_2_[Cr_2_O_7_]·H_2_O (Moon *et al.*, 2017 ▸) and *cis*-[Cr(NCS)(C_10_H_24_N_4_)(*μ*-NCS)ZnCl_3_] (Moon & Choi, 2021 ▸) adopt the *cis*-V conformation.

## Synthesis and crystallization   

The commercially available free ligand cyclam (98%), chromium(III) chloride hexa­hydrate (98%) and sodium bromide (99%) were obtained from Sigma-Aldrich and used as provided. All other chemicals were purchased from commercial sources and used without further purification. The starting material, *cis*-[Cr(NCS)_2_(cyclam)]SCN, was prepared as previously described (Ferguson & Tobe, 1970 ▸). For crystallization of (I), *cis*-[Cr(NCS)_2_(cyclam)]SCN (0.006 g) was dissolved in 5 mL of tetra­hydro­furan at 343 K and the solution filtrated. The filtrate was added to 2 mL of water containing 0.13 g of solid NaBr. The resulting solution was evaporated slowly at room temperature until the formation of crystals suitable for X-ray structural analysis. The obtained needle-like orange crystals of (I) were washed with small amounts of 2-propanol and dried in air before collecting the synchrotron data.

## Refinement   

Crystal data, data collection and structure refinement details are summarized in Table 2 ▸. All H atoms were placed in geometrically idealized positions and constrained to ride on their parent atoms, with C—H = 0.99 Å and N—H = 1.00 Å, and with *U*_iso_(H) values of 1.2*U*_eq_ of the parent atoms.

## Supplementary Material

Crystal structure: contains datablock(s) I. DOI: 10.1107/S2056989021001055/wm5598sup1.cif

Structure factors: contains datablock(s) I. DOI: 10.1107/S2056989021001055/wm5598Isup2.hkl

CCDC reference: 2059465

Additional supporting information:  crystallographic information; 3D view; checkCIF report

## Figures and Tables

**Table 1:** Hydrogen-bond geometry (Å, °)

*D*—H⋯*A*	*D*—H	H⋯*A*	*D*⋯*A*	*D*—H⋯*A*
N1—H1⋯Br1	1.00	2.33	3.327 (3)	177
N2—H2⋯Br1^i^	1.00	2.45	3.352 (3)	150
N3—H3⋯Br1	1.00	2.43	3.389 (3)	161
N4—H4⋯S1^ii^	1.00	2.47	3.410 (3)	156

Symmetry codes: (i) x, y-1, z; (ii) -x+1, y+{\script{1\over 2}}, -z+{\script{3\over 2}}.

**Table 2:** Experimental details

Crystal data	
Chemical formula	[Cr(NCS)_2_(C_10_H_24_N_4_)]Br
*M*_r_	448.40
Crystal system, space group	Monoclinic, *P*2_1_/*c*
Temperature (K)	173
*a*, *b*, *c* (Å)	10.880 (2), 7.7310 (15), 22.161 (4)
β (°)	91.65 (3)
*V* (Å^3^)	1863.3 (6)
*Z*	4
Radiation type	Synchrotron, λ = 0.610 Å
μ (mm^−1^)	1.98
Crystal size (mm)	0.03 × 0.01 × 0.01

Data collection	
Diffractometer	ADSC Q210 CCD area detector
Absorption correction	Empirical (using intensity measurements) (*HKL3000sm *SCALEPACK**; Otwinowski *et al.*, 2003 ▸)
*T*_min_, *T*_max_	0.904, 1.000
No. of measured, independent and observed [*I* > 2σ(*I*)] reflections	18069, 5185, 2901
*R*_int_	0.092
(sin θ/λ)_max_ (Å^−1^)	0.693

Refinement	
*R*[*F* ^2^ > 2σ(*F* ^2^)], *wR*(*F* ^2^), *S*	0.043, 0.100, 0.87
No. of reflections	5185
No. of parameters	200
H-atom treatment	H-atom parameters constrained
Δρ_max_, Δρ_min_ (e Å^−3^)	0.85, −0.79

Computer programs: *PAL BL2D-SMDC* (Shin *et al.*, 2016 ▸), *HKL3000sm* (Otwinowski *et al.*, 2003 ▸), *SHELXT2018* (Sheldrick, 2015*a* ▸), *SHELXL2018* (Sheldrick, 2015*b* ▸), *DIAMOND 4* (Putz & Brandenburg, 2014 ▸) and *publCIF* (Westrip, 2010 ▸).

## supplementary crystallographic information

### Crystal data

**Table:**

[Cr(NCS)_2_(C_10_H_24_N_4_)]Br	*F*(000) = 916
*M**_r_* = 448.40	*D*_x_ = 1.598 Mg m^−^^3^
Monoclinic, *P*2_1_/*c*	Synchrotron radiation, λ = 0.610 Å
*a* = 10.880 (2) Å	Cell parameters from 49610 reflections
*b* = 7.7310 (15) Å	θ = 0.4–33.7°
*c* = 22.161 (4) Å	µ = 1.98 mm^−^^1^
β = 91.65 (3)°	*T* = 173 K
*V* = 1863.3 (6) Å^3^	Needle, orange
*Z* = 4	0.03 × 0.01 × 0.01 mm

### Data collection

**Table:**

ADSC Q210 CCD area detector diffractometer	2901 reflections with *I* > 2σ(*I*)
Radiation source: PLSII 2D bending magnet	*R*_int_ = 0.092
ω scan	θ_max_ = 25.0°, θ_min_ = 2.3°
Absorption correction: empirical (using intensity measurements) (*HKL3000sm Scalepack*; Otwinowski *et al.*, 2003)	*h* = −15→15
*T*_min_ = 0.904, *T*_max_ = 1.000	*k* = −10→10
18069 measured reflections	*l* = −30→30
5185 independent reflections	

### Refinement

**Table:**

Refinement on *F*^2^	Hydrogen site location: inferred from neighbouring sites
Least-squares matrix: full	H-atom parameters constrained
*R*[*F*^2^ > 2σ(*F*^2^)] = 0.043	*w* = 1/[σ^2^(*F*_o_^2^) + (0.0455*P*)^2^] where *P* = (*F*_o_^2^ + 2*F*_c_^2^)/3
*wR*(*F*^2^) = 0.100	(Δ/σ)_max_ = 0.001
*S* = 0.87	Δρ_max_ = 0.85 e Å^−^^3^
5185 reflections	Δρ_min_ = −0.79 e Å^−^^3^
200 parameters	Extinction correction: SHELXL2016/6 (Sheldrick 2015b), Fc^*^=kFc[1+0.001xFc^2^λ^3^/sin(2θ)]^-1/4^
0 restraints	Extinction coefficient: 0.0180 (9)

### Special details

**Table:**

Geometry. All esds (except the esd in the dihedral angle between two l.s. planes) are estimated using the full covariance matrix. The cell esds are taken into account individually in the estimation of esds in distances, angles and torsion angles; correlations between esds in cell parameters are only used when they are defined by crystal symmetry. An approximate (isotropic) treatment of cell esds is used for estimating esds involving l.s. planes.

### Fractional atomic coordinates and isotropic or equivalent isotropic displacement parameters (Å^2^)

**Table:**

	*x*	*y*	*z*	*U*_iso_*/*U*_eq_	
Cr1	0.27993 (5)	0.35081 (6)	0.63702 (2)	0.02286 (14)	
S1	0.31333 (9)	0.00278 (12)	0.81190 (4)	0.0375 (2)	
S2	0.64505 (11)	0.06790 (15)	0.58036 (5)	0.0566 (3)	
N1	0.1153 (3)	0.4392 (3)	0.67081 (12)	0.0279 (6)	
H1	0.097582	0.553841	0.651585	0.033*	
N2	0.1644 (3)	0.2145 (3)	0.57803 (12)	0.0282 (6)	
H2	0.163030	0.093399	0.593809	0.034*	
N3	0.2950 (3)	0.5456 (3)	0.57286 (12)	0.0319 (7)	
H3	0.216688	0.613469	0.572416	0.038*	
N4	0.3741 (3)	0.5269 (3)	0.69205 (12)	0.0312 (6)	
H4	0.460852	0.484332	0.694735	0.037*	
N5	0.2805 (3)	0.1671 (3)	0.70104 (13)	0.0351 (7)	
N6	0.4346 (3)	0.2501 (4)	0.60735 (12)	0.0332 (7)	
C1	0.0176 (3)	0.3171 (4)	0.64907 (15)	0.0333 (8)	
H1A	−0.064580	0.369151	0.654333	0.040*	
H1AB	0.022153	0.208318	0.672605	0.040*	
C2	0.0369 (3)	0.2801 (4)	0.58327 (16)	0.0317 (8)	
H2A	−0.022924	0.192519	0.568261	0.038*	
H2AB	0.025377	0.386957	0.559114	0.038*	
C3	0.2012 (4)	0.2002 (4)	0.51429 (15)	0.0379 (9)	
H3A	0.138689	0.131585	0.491471	0.045*	
H3AB	0.280293	0.137056	0.512905	0.045*	
C4	0.2153 (4)	0.3746 (5)	0.48384 (15)	0.0413 (9)	
H4A	0.136301	0.437517	0.486127	0.050*	
H4AB	0.230961	0.354800	0.440602	0.050*	
C5	0.3170 (4)	0.4903 (5)	0.50993 (15)	0.0405 (9)	
H5A	0.396127	0.427129	0.509024	0.049*	
H5AB	0.324199	0.594165	0.484161	0.049*	
C6	0.3960 (4)	0.6632 (4)	0.59462 (17)	0.0393 (9)	
H6A	0.392736	0.773210	0.571775	0.047*	
H6AB	0.476836	0.608305	0.588356	0.047*	
C7	0.3797 (4)	0.6977 (4)	0.66092 (17)	0.0402 (9)	
H7A	0.449580	0.766556	0.677480	0.048*	
H7AB	0.302857	0.763365	0.666944	0.048*	
C8	0.3351 (4)	0.5454 (5)	0.75602 (15)	0.0391 (9)	
H8A	0.386361	0.635299	0.776192	0.047*	
H8AB	0.350532	0.434806	0.777454	0.047*	
C9	0.2007 (4)	0.5935 (5)	0.76167 (16)	0.0414 (9)	
H9A	0.184689	0.612776	0.804899	0.050*	
H9AB	0.186265	0.704638	0.740453	0.050*	
C10	0.1081 (3)	0.4631 (5)	0.73720 (15)	0.0371 (9)	
H10A	0.122623	0.350514	0.757466	0.044*	
H10B	0.024273	0.502178	0.746855	0.044*	
C11	0.2947 (3)	0.0968 (4)	0.74715 (15)	0.0291 (8)	
C12	0.5225 (3)	0.1731 (4)	0.59660 (14)	0.0322 (8)	
Br1	0.05786 (4)	0.81437 (4)	0.60207 (2)	0.04372 (14)	

### Atomic displacement parameters (Å^2^)

**Table:**

	*U*^11^	*U*^22^	*U*^33^	*U*^12^	*U*^13^	*U*^23^
Cr1	0.0254 (3)	0.0205 (3)	0.0227 (3)	0.0034 (2)	0.0017 (2)	−0.0016 (2)
S1	0.0398 (6)	0.0410 (5)	0.0315 (5)	−0.0035 (4)	−0.0011 (4)	0.0085 (4)
S2	0.0529 (7)	0.0679 (7)	0.0496 (6)	0.0331 (6)	0.0104 (5)	−0.0055 (6)
N1	0.0307 (16)	0.0231 (13)	0.0300 (15)	0.0028 (12)	0.0052 (12)	0.0012 (12)
N2	0.0310 (16)	0.0214 (13)	0.0321 (15)	0.0021 (11)	−0.0007 (12)	0.0011 (12)
N3	0.0316 (17)	0.0328 (15)	0.0315 (15)	−0.0027 (12)	0.0045 (13)	0.0049 (13)
N4	0.0310 (17)	0.0294 (14)	0.0329 (15)	0.0048 (12)	−0.0017 (12)	−0.0063 (13)
N5	0.0406 (18)	0.0292 (15)	0.0355 (16)	0.0079 (13)	0.0023 (13)	0.0050 (14)
N6	0.0339 (18)	0.0359 (15)	0.0298 (16)	0.0057 (14)	0.0038 (13)	−0.0070 (13)
C1	0.0265 (19)	0.0294 (17)	0.044 (2)	−0.0025 (15)	0.0071 (15)	0.0013 (16)
C2	0.0279 (19)	0.0262 (17)	0.041 (2)	−0.0006 (14)	−0.0041 (15)	0.0010 (15)
C3	0.043 (2)	0.0379 (19)	0.0327 (19)	0.0042 (17)	−0.0026 (16)	−0.0077 (17)
C4	0.051 (3)	0.048 (2)	0.0249 (18)	0.0020 (19)	0.0015 (17)	0.0036 (17)
C5	0.046 (2)	0.046 (2)	0.0294 (19)	−0.0014 (19)	0.0067 (17)	0.0103 (17)
C6	0.036 (2)	0.0305 (18)	0.052 (2)	−0.0091 (16)	0.0008 (17)	0.0050 (17)
C7	0.035 (2)	0.0299 (18)	0.056 (2)	−0.0026 (16)	−0.0036 (17)	−0.0073 (18)
C8	0.042 (2)	0.041 (2)	0.034 (2)	0.0043 (17)	−0.0012 (17)	−0.0133 (17)
C9	0.044 (2)	0.044 (2)	0.036 (2)	0.0107 (18)	0.0011 (17)	−0.0163 (18)
C10	0.033 (2)	0.045 (2)	0.0335 (19)	0.0047 (17)	0.0093 (15)	−0.0053 (17)
C11	0.031 (2)	0.0224 (16)	0.0340 (19)	0.0013 (14)	0.0028 (15)	0.0008 (15)
C12	0.041 (2)	0.0298 (17)	0.0259 (17)	0.0044 (16)	0.0040 (15)	−0.0017 (15)
Br1	0.0452 (2)	0.02097 (17)	0.0642 (3)	0.00171 (15)	−0.01224 (18)	0.00375 (17)

### Geometric parameters (Å, º)

**Table:**

Cr1—N6	1.984 (3)	C1—H1AB	0.9900
Cr1—N5	2.007 (3)	C2—H2A	0.9900
Cr1—N2	2.075 (3)	C2—H2AB	0.9900
Cr1—N1	2.076 (3)	C3—C4	1.518 (5)
Cr1—N4	2.078 (3)	C3—H3A	0.9900
Cr1—N3	2.081 (3)	C3—H3AB	0.9900
S1—C11	1.616 (3)	C4—C5	1.524 (5)
S2—C12	1.611 (4)	C4—H4A	0.9900
N1—C10	1.487 (4)	C4—H4AB	0.9900
N1—C1	1.492 (4)	C5—H5A	0.9900
N1—H1	1.0000	C5—H5AB	0.9900
N2—C3	1.484 (4)	C6—C7	1.509 (5)
N2—C2	1.485 (4)	C6—H6A	0.9900
N2—H2	1.0000	C6—H6AB	0.9900
N3—C5	1.485 (4)	C7—H7A	0.9900
N3—C6	1.495 (4)	C7—H7AB	0.9900
N3—H3	1.0000	C8—C9	1.517 (5)
N4—C7	1.492 (4)	C8—H8A	0.9900
N4—C8	1.498 (4)	C8—H8AB	0.9900
N4—H4	1.0000	C9—C10	1.514 (5)
N5—C11	1.164 (4)	C9—H9A	0.9900
N6—C12	1.157 (4)	C9—H9AB	0.9900
C1—C2	1.507 (5)	C10—H10A	0.9900
C1—H1A	0.9900	C10—H10B	0.9900
			
N6—Cr1—N5	88.35 (12)	H2A—C2—H2AB	108.5
N6—Cr1—N2	95.53 (11)	N2—C3—C4	113.0 (3)
N5—Cr1—N2	94.33 (11)	N2—C3—H3A	109.0
N6—Cr1—N1	175.90 (11)	C4—C3—H3A	109.0
N5—Cr1—N1	87.88 (11)	N2—C3—H3AB	109.0
N2—Cr1—N1	83.16 (11)	C4—C3—H3AB	109.0
N6—Cr1—N4	92.48 (12)	H3A—C3—H3AB	107.8
N5—Cr1—N4	93.29 (12)	C3—C4—C5	115.7 (3)
N2—Cr1—N4	169.10 (11)	C3—C4—H4A	108.4
N1—Cr1—N4	89.34 (11)	C5—C4—H4A	108.4
N6—Cr1—N3	88.49 (12)	C3—C4—H4AB	108.4
N5—Cr1—N3	175.08 (12)	C5—C4—H4AB	108.4
N2—Cr1—N3	89.72 (11)	H4A—C4—H4AB	107.4
N1—Cr1—N3	95.39 (11)	N3—C5—C4	113.0 (3)
N4—Cr1—N3	83.08 (11)	N3—C5—H5A	109.0
C10—N1—C1	109.9 (3)	C4—C5—H5A	109.0
C10—N1—Cr1	117.9 (2)	N3—C5—H5AB	109.0
C1—N1—Cr1	106.85 (19)	C4—C5—H5AB	109.0
C10—N1—H1	107.2	H5A—C5—H5AB	107.8
C1—N1—H1	107.2	N3—C6—C7	108.4 (3)
Cr1—N1—H1	107.2	N3—C6—H6A	110.0
C3—N2—C2	112.3 (3)	C7—C6—H6A	110.0
C3—N2—Cr1	117.5 (2)	N3—C6—H6AB	110.0
C2—N2—Cr1	109.14 (19)	C7—C6—H6AB	110.0
C3—N2—H2	105.6	H6A—C6—H6AB	108.4
C2—N2—H2	105.6	N4—C7—C6	107.5 (3)
Cr1—N2—H2	105.6	N4—C7—H7A	110.2
C5—N3—C6	109.9 (3)	C6—C7—H7A	110.2
C5—N3—Cr1	116.8 (2)	N4—C7—H7AB	110.2
C6—N3—Cr1	106.9 (2)	C6—C7—H7AB	110.2
C5—N3—H3	107.6	H7A—C7—H7AB	108.5
C6—N3—H3	107.6	N4—C8—C9	113.7 (3)
Cr1—N3—H3	107.6	N4—C8—H8A	108.8
C7—N4—C8	111.7 (3)	C9—C8—H8A	108.8
C7—N4—Cr1	109.5 (2)	N4—C8—H8AB	108.8
C8—N4—Cr1	118.0 (2)	C9—C8—H8AB	108.8
C7—N4—H4	105.5	H8A—C8—H8AB	107.7
C8—N4—H4	105.5	C10—C9—C8	116.1 (3)
Cr1—N4—H4	105.5	C10—C9—H9A	108.3
C11—N5—Cr1	161.6 (3)	C8—C9—H9A	108.3
C12—N6—Cr1	169.9 (3)	C10—C9—H9AB	108.3
N1—C1—C2	108.3 (3)	C8—C9—H9AB	108.3
N1—C1—H1A	110.0	H9A—C9—H9AB	107.4
C2—C1—H1A	110.0	N1—C10—C9	112.5 (3)
N1—C1—H1AB	110.0	N1—C10—H10A	109.1
C2—C1—H1AB	110.0	C9—C10—H10A	109.1
H1A—C1—H1AB	108.4	N1—C10—H10B	109.1
N2—C2—C1	107.3 (3)	C9—C10—H10B	109.1
N2—C2—H2A	110.3	H10A—C10—H10B	107.8
C1—C2—H2A	110.3	N5—C11—S1	178.8 (3)
N2—C2—H2AB	110.3	N6—C12—S2	178.9 (3)
C1—C2—H2AB	110.3		
			
C10—N1—C1—C2	−173.5 (3)	C5—N3—C6—C7	−172.6 (3)
Cr1—N1—C1—C2	−44.5 (3)	Cr1—N3—C6—C7	−44.9 (3)
C3—N2—C2—C1	−170.8 (3)	C8—N4—C7—C6	−169.6 (3)
Cr1—N2—C2—C1	−38.6 (3)	Cr1—N4—C7—C6	−36.9 (3)
N1—C1—C2—N2	55.7 (3)	N3—C6—C7—N4	54.7 (4)
C2—N2—C3—C4	68.6 (4)	C7—N4—C8—C9	71.2 (4)
Cr1—N2—C3—C4	−59.2 (4)	Cr1—N4—C8—C9	−57.1 (4)
N2—C3—C4—C5	64.2 (4)	N4—C8—C9—C10	62.9 (4)
C6—N3—C5—C4	−178.1 (3)	C1—N1—C10—C9	−176.9 (3)
Cr1—N3—C5—C4	59.9 (4)	Cr1—N1—C10—C9	60.4 (3)
C3—C4—C5—N3	−64.9 (4)	C8—C9—C10—N1	−64.3 (4)

### Hydrogen-bond geometry (Å, º)

**Table:**

*D*—H···*A*	*D*—H	H···*A*	*D*···*A*	*D*—H···*A*
N1—H1···Br1	1.00	2.33	3.327 (3)	177
N2—H2···Br1^i^	1.00	2.45	3.352 (3)	150
N3—H3···Br1	1.00	2.43	3.389 (3)	161
N4—H4···S1^ii^	1.00	2.47	3.410 (3)	156

Symmetry codes: (i) *x*, *y*−1, *z*; (ii) −*x*+1, *y*+1/2, −*z*+3/2.
